# Laboratory Investigation of Packing Characteristics and Mechanical Performance of Aggregate Blend

**DOI:** 10.3390/ma18091953

**Published:** 2025-04-25

**Authors:** Weixiao Yu, Yun Li, Zhipeng Liang, Jiaxi Wu, Sudi Wang, Yinghao Miao

**Affiliations:** 1 National Center for Materials Service Safety, University of Science and Technology Beijing, Beijing 100083, China; yuweixiao@xs.ustb.edu.cn (W.Y.); wujiaxi94520@163.com (J.W.); 2 Beijing Transportation Infrastructure Construction Project Management Center, Beijing 100053, China; liyun88930@foxmail.com (Y.L.); zhipengliang@126.com (Z.L.); 3 Research Institute of Highway Ministry of Transport, Beijing 100088, China

**Keywords:** aggregate blend, packing characteristics, mechanical performance, California bearing ratio

## Abstract

Aggregates are the main material forming the skeleton structure of asphalt mixtures and are of great importance to resist external load for asphalt pavement. This study analyzed the packing characteristics and mechanical performance of aggregate blend to provide a reference for improving the bearing capacity of asphalt mixtures. The single-size, two-size, and multi-size aggregate blends were chosen to conduct the laboratory packing and California bearing ratio (CBR) tests. Six particle sizes were selected to design the single-size aggregate blends. Six size combinations were included and various mass ratios were considered for each size combination in the two-size aggregate blends. The multi-size aggregate blends were designed through the gradually filling method according to stone matrix asphalt with a nominal maximum particle size (NMPS) of 16 mm (SMA16) and dense asphalt concrete with an NMPS of 26.5 mm (AC25). The packing characteristics of the blends were quantified by the air voids and the percentage of contribution to the packing volume (*PCPV*). The mechanical performance of the blends was analyzed by the CBR value. The relationship between packing characteristics and mechanical performance was explored by data fitting. The results showed that the particle size and the size ratio have an effect on the packing characteristics and mechanical performance of aggregate blend. The smaller the particle size, the larger the air void of the blend. The blends composed of larger particles have better load bearing capacity than those composed of smaller particles. The larger the particle size ratio, the greater the air void of the blend and the weaker the load bearing capacity. The particles smaller than 1.18 mm and those smaller than 0.3 mm in AC25 mainly play a role in filling the voids and have little contribution to the load bearing. There is a certain correlation between the packing characteristics and mechanical performance of aggregate blend.

## 1. Introduction

Aggregates are the main material forming the skeleton structure of asphalt mixture and resisting external load [1]. Aggregate packing characteristics have a significant influence on the skeleton structure and the anti-deformation ability of asphalt mixtures [2,3]. Enhancing the packing degree of aggregate particles contributes to improving the engineering properties of paving materials [4]. Asphalt mixture is a multiphase material composed of coarse aggregates, fine aggregates, fillers, asphalt binders, and air voids. The volume parameters of asphalt mixtures are determined by the packed aggregates and usually applied to instruct the composition design [5]. Therefore, it is necessary to investigate the packing characteristics and mechanical performance of aggregate blend to provide a reference for improving the performance and optimizing the design method for asphalt mixture.

Traditional asphalt mixture gradation design is mainly based on the power function curve [6]. This method can achieve the densest packing and maximum density of aggregates, but it is unable to ensure the anti-rutting performance of asphalt mixtures. Several researchers attempted to conduct the design of aggregate blend by packing test and macroscopic volume parameters. The Bailey method concluded that the critical size of coarse aggregate and fine aggregate is 0.22 times of the nominal maximum aggregate size (NMAS) through the geometric packing model of spheres [7]. Weymouth [8] proposed particle interfering theory and developed the calculation method of aggregate gradation for cement concrete to improve workability. Kim et al. [9,10,11] proposed a dominant aggregate size range (DASR) model to evaluate the composition features of aggregate blends. The method suggested that the voids formed by DASR particles should be filled by small particles and asphalt mastics. Guarin [12] determined the voids between DASR particles based on particle packing theory and proposed an interference coefficient and interference particle size range. Miao et al. [13] defined the percentage of contribution to the packing volume (PCPV) index to quantify the packing function of particles in packed aggregate blend. They also analyzed the effect of particle size and morphology on packing characteristics of aggregates [14]. The results showed that the packing function of particles is decided by both particle size and the composition of particles with different sizes. In summary, the above research mainly conducts geometric model-based theoretical analysis, lacking attention to the laboratory performance of aggregate blends.

The digital image processing (DIP) technique provides another way to evaluate the packing characteristics of aggregate blend. Shi et al. [15] obtained the meso-structural evaluation indicators of coarse aggregate main skeleton and asphalt mortar interference by DIP technique. The results showed that the number of contact points and pore cells within the asphalt mixture increase, interference coefficient decreases, and skeleton rate increases gradually under the action of loads. Cai et al. [16] characterized the asphalt mixture skeleton under various cycles of compaction repetitions by coarse aggregate contact points, aggregate inclination, and some other indexes. Xing et al. [17] proposed a skeleton filling system evaluation method of asphalt mixture based on the compressible packing model and industrial computed tomography (CT). Wang et al. [18] found that gradation types have a significant impact on average contact number and internal structure index of asphalt mixtures. Sun et al. [19] evaluated the homogeneity of asphalt mixture based on a digital image processing technique and stereology theory. The results indicated that a decrease in the nominal maximum aggregate size and a finer aggregate gradation can improve the homogeneity of asphalt mixtures. Moreover, some other indicators describing aggregate contact can also be used to evaluate the packing characteristic, such as contact point number [20,21], co-ordination number [22], and the distribution of air voids [23]. In summary, it is feasible to analyze the packing characteristics using DIP technique. However, the method for obtaining the sectional image of asphalt mixture is complicated and costly. In addition, the analysis of packing characteristics is not comprehensive enough.

The discrete element method (DEM) is also usually used to study the packing characteristics of aggregate blend. Xing et al. [24] studied the skeleton filling system of asphalt mixture and found that the variation coefficient of contact force of asphalt mortar with low filling coefficient is larger and the localization phenomenon is more serious. Shen et al. [25] investigated the effect of particle size on the packing of aggregate particles based on DEM method. The results showed that aggregate size distribution played a significant role in the packing characteristics, affecting both volumetrics and the contact characteristics of a packed structure. Chen et al. [26] analyzed the voids content of unbound aggregate blend under dry-rodded condition to evaluate the development of aggregate packing in porous asphalt mixture. Miao et al. [27] investigated the function of different-sized particles in packed aggregate blend using DEM method and found that the main function conversion particle size is 2.36 mm for SMA-16 and 4.75 mm for AC-25. Kusumawardani et al. [28] studied the packing conditions of unbound aggregate blends and porous asphalt mixture by nine aggregate gradations with variations in 2.36–4.75 mm and 6.3–9.5 mm fractions. The results showed that 6.3–9.5 mm aggregates have little influence on the aggregate packing structure, while 2.36–4.75 mm aggregates have obvious influence on it. In summary, many researchers mainly focus on the packing characteristics of aggregate blend and the factors affecting packing characteristics from macroscopic and mesoscopic views (macroscopic and mesoscopic views refer to the performance of mixtures and the features of aggregates in mixtures, respectively), which contributes to fully understanding the load transfer mechanism. However, little attention has been paid to the relationship between the packing characteristics and mechanical characteristics of aggregate blends.

The objective of this study is to analyze the effect of packing characteristics of aggregate blend on its mechanical performance. The single-size, two-size, and multi-size aggregate blends were selected to conduct the packing and California bearing ratio (CBR) tests. The packing characteristics of the blends were quantified by the air voids and the packing volume. The mechanical performance of the blends was analyzed by the CBR value. The relationship between packing characteristics and mechanical performance were explored by data fitting. This study can provide a reference for improving the performance and optimizing the design method for asphalt mixture.

## 2. Materials and Methods

### 2.1. Materials

**Figure 1:**
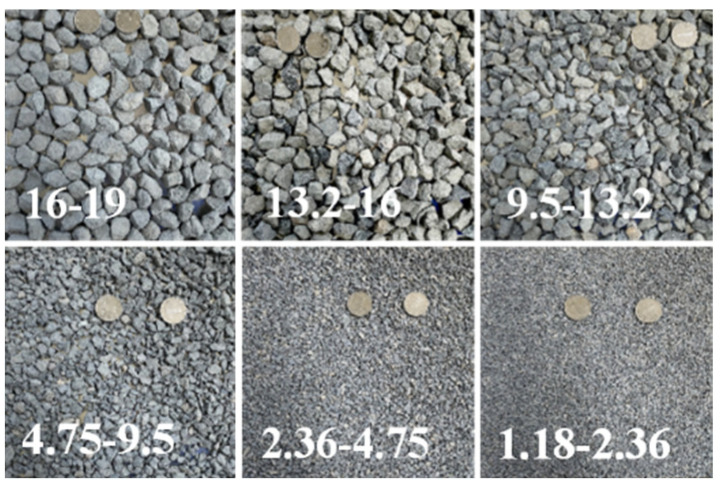
Aggregates with different sizes.

The crushed stone, manufactured sand, and limestone mineral powder were, respectively, selected as coarse aggregates, fine aggregates, and filler to perform the laboratory tests. Figure 1 depicts the selected aggregates. The properties of different-sized aggregates and filler were determined according to the Chinese specification “Test Methods of Aggregate for Highway Engineering (JTG E42-2005)” [29], which is listed in Table 1. It should be noted that the Los Angeles abrasion of coarse aggregates is able to meet the requirements of the Chinese specification “Technical Specification for Construction of Highway Asphalt Pavements (JTG F40-2004)” [30] according to the report provided by the manufacturer, so the Los Angeles abrasion’s range of the specification requirements (≤28%) is only presented in Table 1.

### 2.2. Aggregate Blends

**Figure 2:**
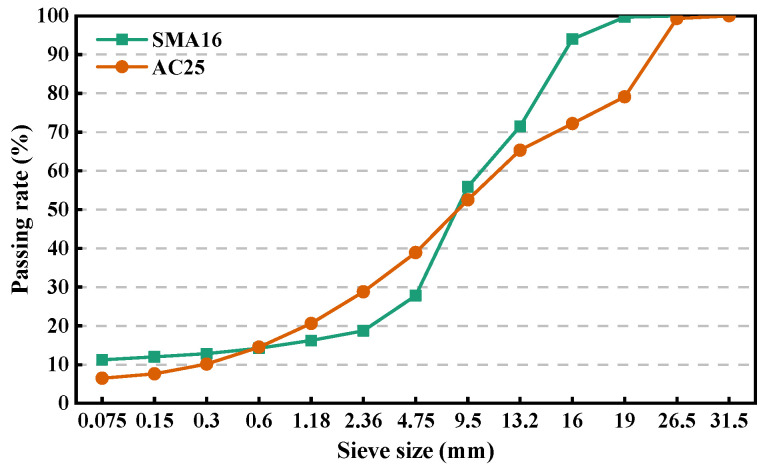
Gradation curves.

The single-size, two-size, and multi-size aggregate blends were employed to conduct the investigation of packing characteristics and mechanical performance. Three parallel tests were performed for each aggregate blend, and the average value of test results was adopted. For the single-size aggregate blends, six sizes were involved. For the two-size aggregate blends, six size combinations were included and various mass ratios were considered for each size combination to understand the influence of aggregate composition, particle size, and size ratio. To explore the contribution of specific size particles to the packing and mechanical performance, the multi-size aggregate blends were designed through the gradually filling method based on the blend with specific gradation, which has been detailed described in the reference [13], and the composition ratios were determined according to two typical gradations, namely stone matrix asphalt with a nominal maximum particle size (NMPS) of 16 mm (SMA16) and dense asphalt concrete with an NMPS of 26.5 mm (AC25). The reasons for selecting AC25 are as follows: (1) AC25 gradation is commonly used on the lower surface or base course of asphalt pavement; (2) more multi-size aggregate blends can be designed according to the gradation with larger NMPS design, contributing to better analyzing the change in packing characteristic and mechanical performance in the gradually filling process of smaller particles. Figure 2 depicts the gradations of SMA16 and AC25 used in this study. Table 2 lists the information of the designed aggregate blends. Three parallel experiments were performed for each blend and the average values were taken for analysis. For convenience, the particles retained on *s* mm sieve but passing the *s* + 1 mm sieve are denoted as *s* mm particles. It should be noted that *s* + 1 is the smallest sieve size in the sieves larger than *s* mm. For example, if *s* is 2.36, *s* + 1 is 4.75. And 2.36 mm aggregates represent the particles retained on 2.36 mm sieve but passing the 4.75 mm sieve. *A_s_* is used to represent the single-size blend composed of the particles with the size of *s* mm. a + b represents the two-size aggregate blend composed of a mm and b mm particles. *A_s-M_* is employed to represent the multi-size blend composed of particles with a size larger than *s* mm in SMA16 or AC25 gradation. *A* represents the aggregate blend with given gradation.

### 2.3. Packing Test

The packing tests were performed in a cylinder with a height of 170 mm and a diameter of 152 mm by vibration method in accordance with the method of T0309-2005 in the technological specification “Test Methods of Aggregate for Highway Engineering (JTG E42-2005)” of China [29]. The mass of each sample is 3 kg. The vibration time for each aggregate blend is 2 min, the vibration frequency is 50 Hz, and the amplitude is 0.5 mm. For the single-size aggregate blends, the air void was used to evaluate the packing characteristics. For the two-size and multi-size aggregate blends, the air void, the percentage of contribution to the packing volume (PCPV), and the change in air void from *A*_(*s+1*)*-M*_ to *A_s-M_* were selected to characterize the packed aggregates, which are defined as Equations (1–3), respectively [13].

(1) $$V_{a}=\frac{V_{B}^{blend}}{V_{P}^{blend}}$$

(2) $$PCPV_{s\text{-}M}=\frac{V_{p}^{s\text{-}M}}{V_{p}^{A}}$$

(3) $$RV_{s}=V_{a}^{(s+1)\text{-}M}-V_{a}^{s\text{-}M}$$

where *V_a_* is the air void of an aggregate blend; $V_{B}^{blend}$ is the bulk volume of an aggregate blend; $V_{P}^{blend}$ is the packing volume of an aggregate blend; *PCPV_s-M_* is the contribution of *A_s-M_* to the packing volume of *A* (for the two-size blend, *PCPV_s-M_* is the contribution of larger particles to the packing volume of a + b blend); $V_{P}^{s\text{-}M}$ is the packing volume of *A_s-M_* (for the two-size blend, $V_{P}^{s\text{-}M}$ is the packing volume of packed larger particles); $V_{P}^{A}$ is the packing volume of *A* (for the two-size blend, $V_{P}^{A}$ is the packing volume of a + b blend); *RV_s_* is the change in air void after adding *s* mm particles in *A_(s+1)-M_* (for the two-size blend, *RV_s_* is the change in air void after adding smaller particles in larger particles); $V_{a}^{(s+1)\text{-}M}$ is the air void of *A_(s+1)-M_* (for the two-size blend, $V_{a}^{(s+1)\text{-}M}$ is the air void of packed larger particles); and $V_{a}^{s\text{-}M}$ is the air void of *A_s-M_* (for the two-size blend, $V_{a}^{s\text{-}M}$ is the air void of a + b blend).

**Figure 3:**
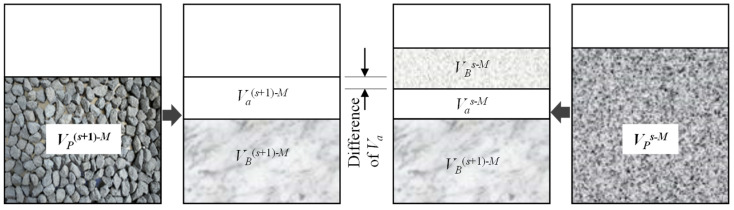
The relationships between $V_{a}^{(s+1)\text{-}M}$, $V_{a}^{s\text{-}M}$, $V_{P}^{(s+1)\text{-}M}$, and $V_{P}^{s\text{-}M}$.

The packing function of aggregate particles in a blend mainly includes skeleton building and air void filling. The addition of *s* mm particles into *A*_(*s*+1)*-M*_ will cause the change in air void content of the blend, and the larger the decrease in the air void content, the better its air void filling function. Therefore, the *RV_s_* can be used to quantify the air void filling contribution of *s* mm particles in a blend. The larger the *RV_s_*, the stronger the air void filling function of *s* mm particles. Figure 3 depicts the relationships between $V_{a}^{(s+1)\text{-}M}$, $V_{a}^{s\text{-}M}$, $V_{P}^{(s+1)\text{-}M}$, and $V_{P}^{s\text{-}M}$.

### 2.4. California Bearing Ratio (CBR) Test

**Figure 4:**
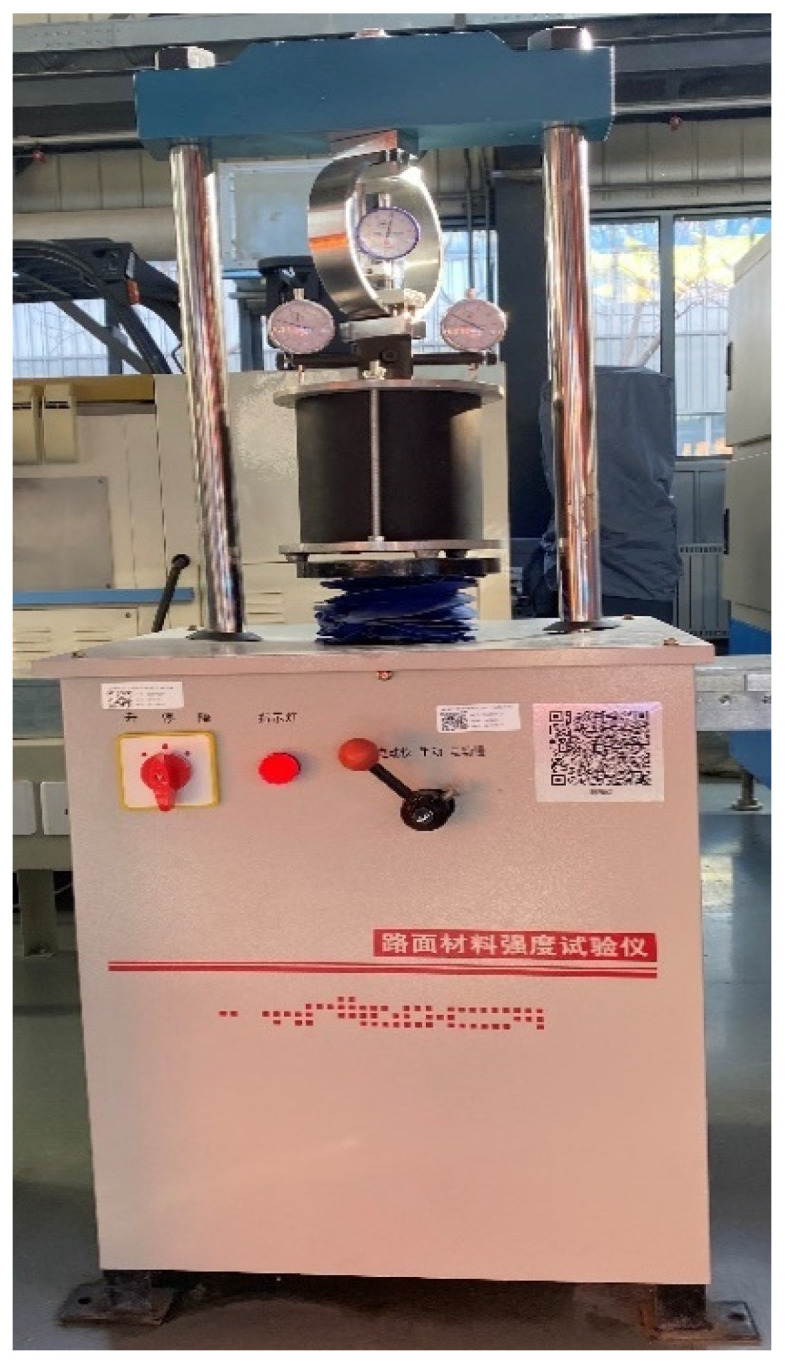
LD-127 pavement material strength tester.

The California bearing ratio (CBR) test is a relatively easy and inexpensive test and has been widely used to characterize the bearing capacity of unbound granular materials in pavement structures [31,32,33]. Although the CBR value provides only a relative strength, it has been considered to provide a reasonable way to assess the material performance [34] and closely related to the stability of asphalt mixture [35]. Therefore, this study adopted the CBR test to assess the mechanical performance of aggregate blend according to Chinese technological specifications ‘‘Test Methods of soils for Highway Engineering (JTG 3430-2020)” [36]. The specific experimental steps are as follows: (1) making CBR specimens according to the vibration method in Section 2.3; (2) soaking the CBR specimens in water for 4 days to make them reach saturation; (3) penetrating into the specimen using a standard indenter with an area of 19.35 cm^2^ of LD-127 pavement material strength tester (as shown in Figure 4) at a speed of 0.1 cm/min; and (4) recording the penetration force per 0.25 cm of penetration depth until it reaches 1.25 cm. The value of CBR is defined as the proportion of the unit pressure to the standard pressure at a certain penetration depth, as shown in Equation (4). The unit pressure is defined as the proportion of the penetration force to the area of the indenter. The standard pressure is the unit pressure of high-quality standard crushed stones, which is listed in Table 3. It should be noted that the CBR value is calculated at a penetration depth of 0.25 cm. However, if the CBR value at the penetration of 0.25 cm is less than that at the penetration of 0.5 cm, the latter should be used as the test result.

(4) $$CBR=\frac{P}{P_{i}}\times 100\%$$

where *P* is the unit pressure of aggregate blend at a certain penetration depth, kPa; *P_i_* is the standard pressure at the corresponding penetration depth, kPa.

## 3. Results and Discussion

### 3.1. Packing Characteristics of Aggregate Blend

#### 3.1.1. Single-Size Blends

**Figure 5:**
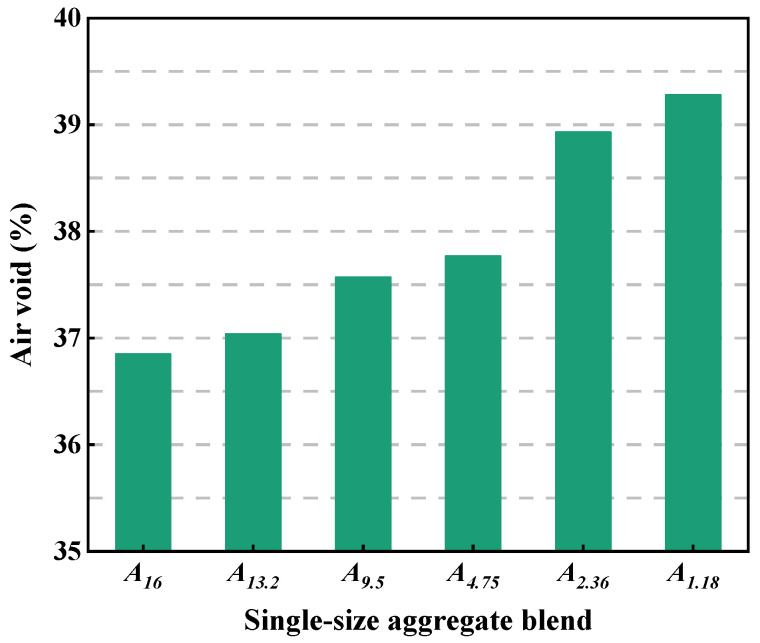
Air voids of single-size aggregate blends.

Figure 5 depicts the air voids of single-size aggregate blends. As can be seen, the air void is between 36% and 40% for the single-size aggregate blend. The particle size has a significant effect on the air void of the blend. The smaller the particle size, the larger the air void of the single-size aggregate blend. This is because the larger aggregates are minimally affected by the surface force and their position can be adjusted more easily on the vibration table compared with the smaller aggregates.

#### 3.1.2. Two-Size Blends

**Figure 6:**
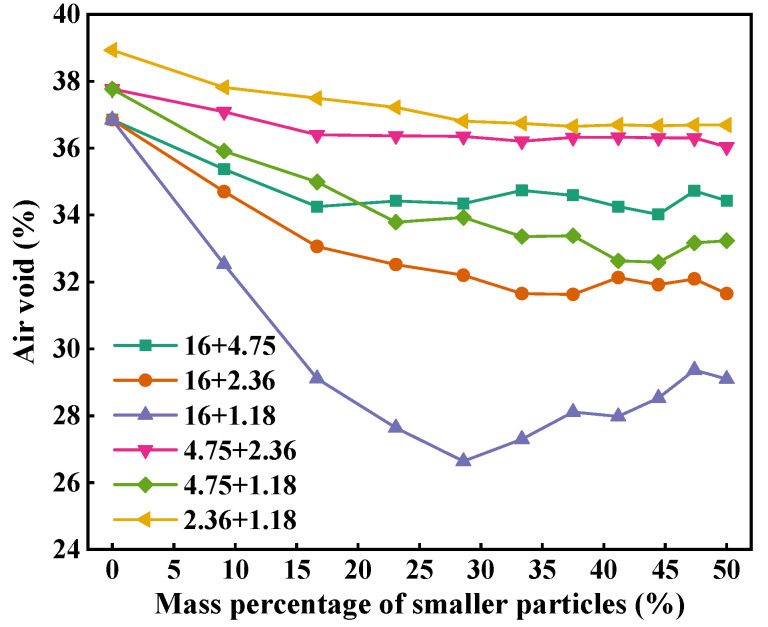
Air voids of two-size aggregate blends.

Figure 6 depicts the air voids of two-size aggregate blends. As can be seen, with the increase in the mass percentage of smaller particles, the air voids of all two-size aggregate blends gradually decrease first and then keep stable, except 16 + 1.18. The air voids of 16 + 1.18 blends decrease first and then gradually increase with increase in 1.18 mm particles. Therefore, the addition of smaller particles in larger particles can reduce the air void of the blend. The air void of 16 + 4.75 is larger than that of 16 + 2.36 and 16 + 1.18, and the air void of 4.75 + 2.36 is greater than that of 4.75 + 1.18 at the same mass percentage of smaller particles. This indicates that particle size ratio has a significant effect on the packing characteristics of the blend. The larger the size ratio, the greater the air void of the blend at the same mass percentage of smaller particles and the faster the decrease in the air void with the increase in the mass percentage of smaller particles. In addition, the air voids of 16 + 4.75, 4.75 + 2.36, and 2.36 + 1.18 follow the order of 2.36 + 1.18 > 4.75 + 2.36 > 16 + 4.75, indicating that the particle size also has a significant effect on the air void of the blend. The smaller the particle size, the larger the air void of the blend.

**Figure 7:**
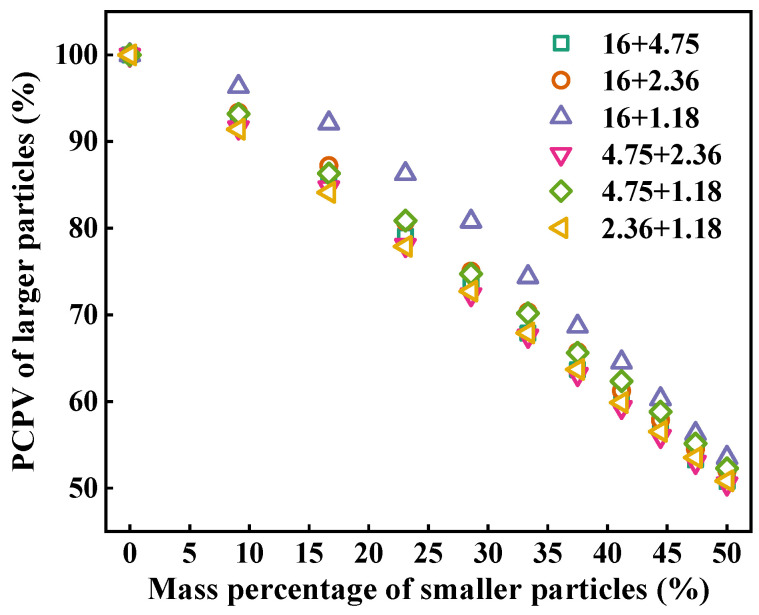
PCPV of larger particles in two-size aggregate blends.

Figure 7 describes the contribution of the larger particles to packing volume in two-size aggregate blends. It can be seen that the PCPV of larger particles shows a decrease trend with the increasing mass percentage of smaller particles in two-size aggregate blend. At the same mass percentage of smaller particles, the PCPV of the larger particles in 16 + 1.18 blends is larger than that in other blends, and that in 16 + 4.75, 16 + 2.36, 4.75 + 2.36, 4.75 + 1.18, and 2.36 + 1.18 blends is almost equal. The difference in the PCPV of larger particles between 16 + 4.75, 16 + 2.36, 4.75 + 2.36, 4.75 + 1.18, and 2.36 + 1.18 blends is less than 3% and that between 16 + 1.18 and other blends is between 2% and 9%. Therefore, only when the particle size ratio is very high will it have an effect on the PCPV of larger particles.

**Figure 8:**
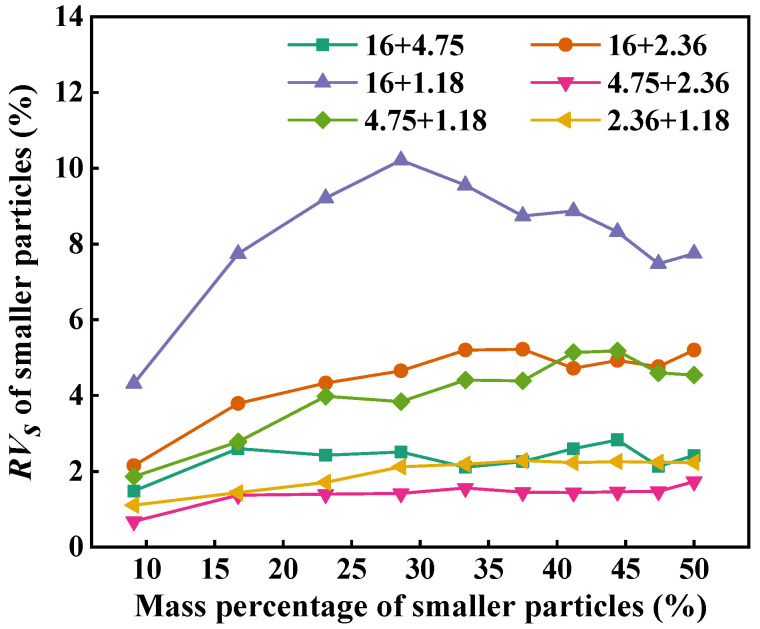
*RV_s_* of smaller particles in two-size aggregate blends.

Figure 8 depicts the *RV_s_* of smaller particles in two-size aggregate blends. As can be seen, the *RV_s_* of the smaller particles shows an increasing trend with the increase in the mass percentage. The *RV_s_* of the smaller particles is greater than 0, indicating that the addition of smaller-sized particles in larger-sized particles can reduce the air voids of the blend. The *RV_s_* of the smaller particles in 16 + 1.18 is larger than that in 16 + 2.36 and 16 + 4.75 at the same mass percentage, which indicates that, the larger the particle size ratio, the greater the air void filling contribution of smaller-sized particles in two-size blends, and the smaller the particle size ratio, the weaker the air void filling contribution of smaller-sized particles.

#### 3.1.3. Multi-Size Blends

**Figure 9:**
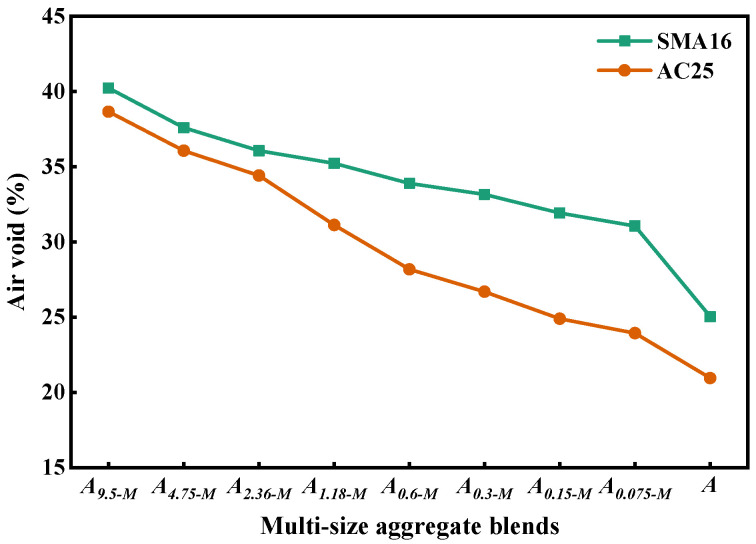
Air void of multi-size aggregate blend.

Figure 9 depicts the air void of multi-size aggregate blend. It can be seen that the air void of *A_(s+1)-M_* gradually decreases with the addition of *s* mm particles in it, indicating that the dense degree of the blend becomes better and the density of aggregate blend gradually increases. The designed *A_s-M_* according to SMA16 has larger air void than that according to AC25, which indicates that the composition ratio of particles with different sizes has a significant effect on the air void of the blend.

**Figure 10:**
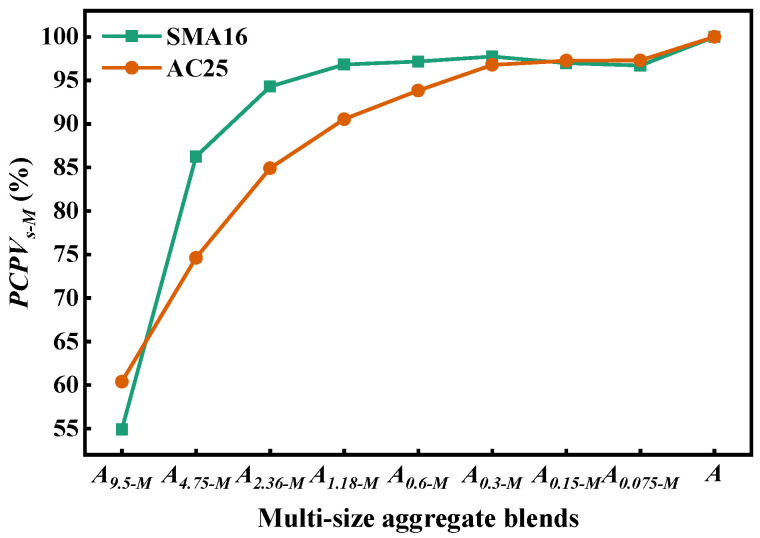
*PCPV_s-M_* of multi-size aggregate blend.

Figure 10 shows the contribution of *A_s-M_* to the packing volume of *A*. As can be seen, the change in *PCPV_s-M_* can be divided into two stages: increasing stages and steady stages. For the designed multi-size blends according to SMA16, the *PCPV_s-M_* will not change when gradually adding the particles with a size smaller than 1.18 mm into *A_s-M_*. For the designed multi-size blends according to AC25, the *PCPV_s-M_* will not change when gradually adding the particles with a size smaller than 0.3 mm into *A_s-M_*. This indicates that particles smaller than 1.18 mm fully fill the voids in SMA16 and thosesmaller than 0.3 mm fully fill the voids in AC25. The *PCPV_s-M_* in SMA-16 grows faster than that in AC25, indicating that the composition ratio of particles with different sizes has an effect on the PCPV of the blend. Moreover, the fillers mainly provide better adhesive behavior between aggregates and asphalt binders.

**Figure 11:**
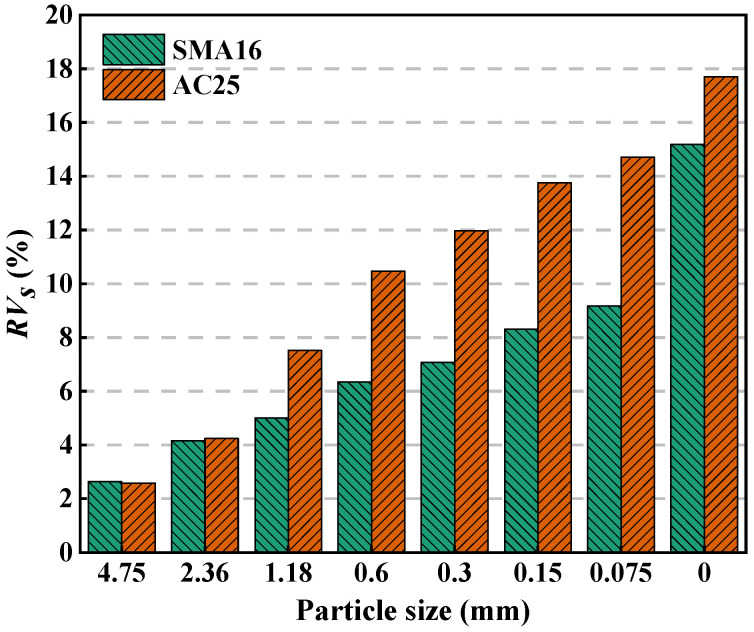
*RV_s_* in multi-size aggregate blend.

Figure 11 depicts the *RV_s_* in multi-size aggregate blend. As can be seen, the smaller the particle, the larger the *RV_s_*. This indicates that smaller particles have higher air void filling contribution. All the *RV_s_* values are greater than 0, which indicates that all particles have air void filling contribution in the corresponding blend. In general, the *RV_s_* in SMA16 is smaller than that in AC25. This indicates that the air voids of the designed multi-size blends according to AC25 decrease more quickly with the addition of smaller particles compared with that of the designed multi-size blends according to SMA16.

### 3.2. Mechanical Performance of Aggregate Blend

#### 3.2.1. Single-Size Blends

**Figure 12:**
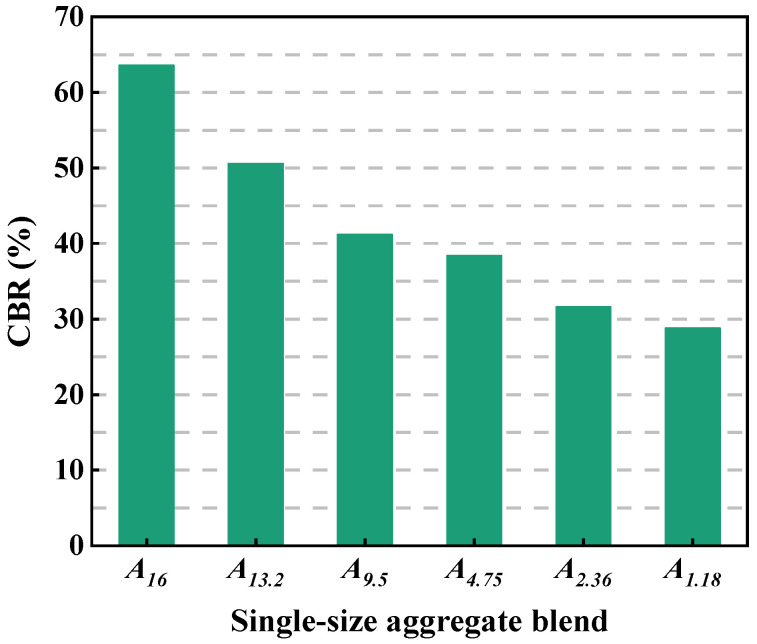
CBR of the single-size aggregate blend.

Figure 12 depicts the CBR of the single-size aggregate blend. As shown in Figure 9, the particle size has an obvious influence on the values of CBR. The larger the particle size, the higher the values of CBR, indicting that larger particles have better bearing capacity and mechanical performance.

#### 3.2.2. Two-Size Blends

**Figure 13:**
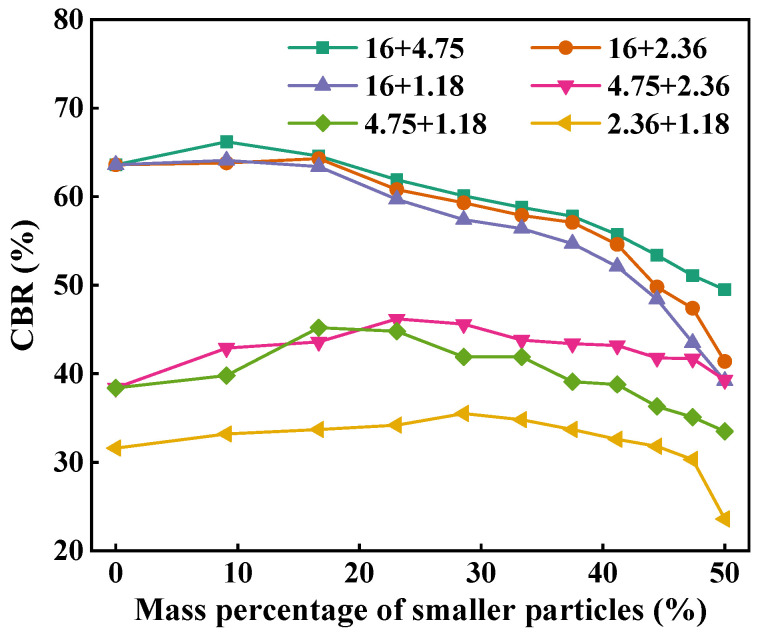
CBR of the two-size aggregate blend.

Figure 13 describes the CBR value of two-size aggregate blend. As can be seen, with the increase in the mass percentage of smaller particles, the CBR value of the blend slightly increases first and then gradually decreases. This indicates that the mechanical performance and bearing capacity of the blend can be improved by properly increasing the smaller particles in larger particles. For the 16 + 1.18 and 16 + 2.36 blends, the CBR values are largest when the mass percentage of the smaller particles is 16.7%. For the 16 + 1.18, 16 + 2.36, and 16 + 4.75 blends, the CBR values decrease significantly when the mass percentage of smaller particles is more than 30%. This is because excessive smaller particles destroy the skeleton structure of larger particles, resulting in the reduction in the bearing capacity of the blend. In general, at the same mass percentage of smaller particles, the CBR values of 16 + 4.75, 16 + 2.36, and 16 + 1.18 blends follow the order of 16 + 4.75 > 16 + 2.36 > 16 + 1.18 and that of 4.75 + 2.36 is larger than that of 4.75 + 1.18. This indicates that size ratio has a significant effect on the mechanical performance of the blends and, the larger the particle size ratio, the weaker the load bearing capacity. The CBR value of 16 + 4.75 is greater than that of 4.75 + 2.36 and that of 2.36 + 1.18 is smallest, indicating that the blends composed of larger particles have better load bearing capacity than those composed of smaller particles.

#### 3.2.3. Multi-Size Blends

**Figure 14:**
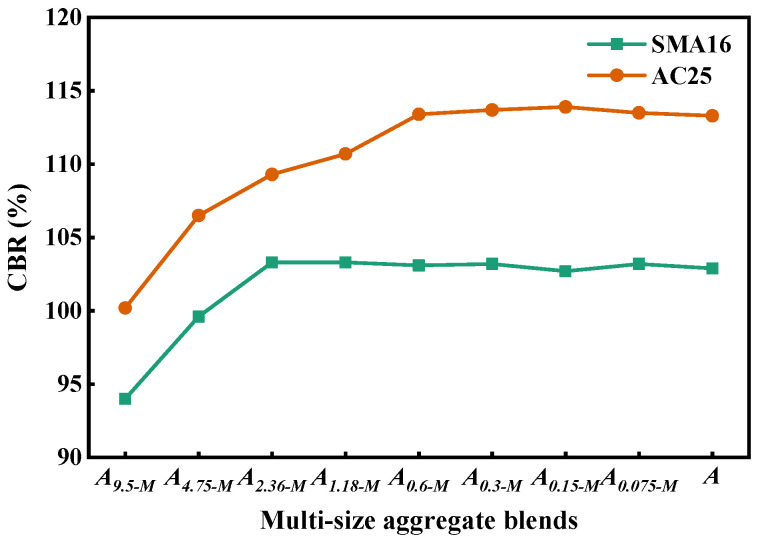
CBR of the multi-size aggregate blend.

Figure 14 depicts the CBR value of the multi-size aggregate blend. It can be seen that the CBR values of the blends increase first and then keep stable with the addition of smaller particles. This indicates that the addition of smaller particles can improve the bearing capacity of mixtures and reduce the crushing of aggregates, which contributes to enhance the stability of asphalt mixtures. For the designed multi-size blends according to SMA16, the CBR will not change when adding particles with a size smaller than 1.18 mm into *A_s-M_*. For the designed multi-size blends according to AC25, the CBR will not change when adding particles with a size smaller than 0.3 mm into *A_s-M_*. The above results indicate that the particles smaller than 1.18 mm in SMA16 and those smaller than 0.3 mm in AC25 mainly play a role to fill the voids and have little contribution to the load bearing. The designed blends according to AC25 have a larger CBR compared with that according to SMA16, indicating that the load bearing capacity of AC25 is better than that of SMA16.

### 3.3. Relationship Between Packing Characteristics and Mechanical Performance

#### 3.3.1. Single-Size Blends

**Figure 15:**
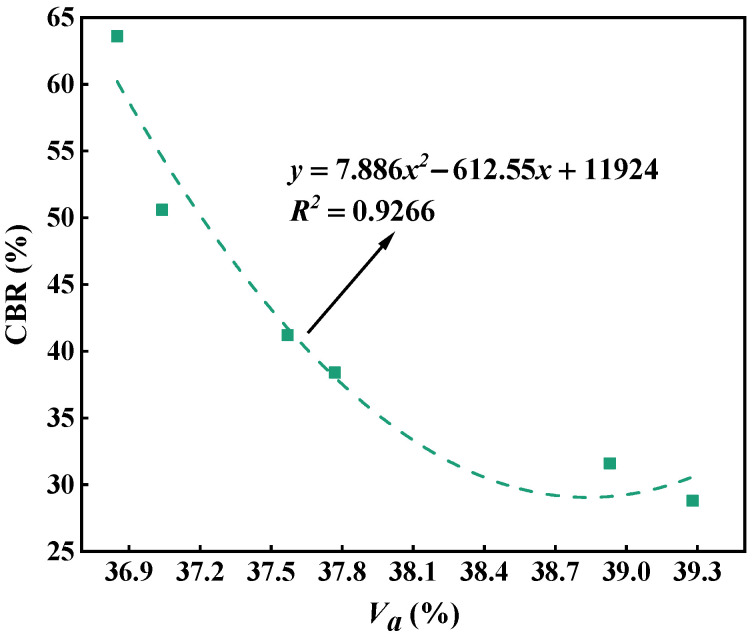
Relationship between the air void and CBR for the single-size aggregate blend.

Figure 15 describes the relationship between the air void and CBR for the single-size aggregate blend. It can be seen that, for the single-size aggregate blend, the larger the air void, the smaller the CBR value, and the air void and CBR shows a significant quadratic relationship. Therefore, the air void of the blend has an effect on the mechanical performance.

#### 3.3.2. Two-Size Blends

**Figure 16:**
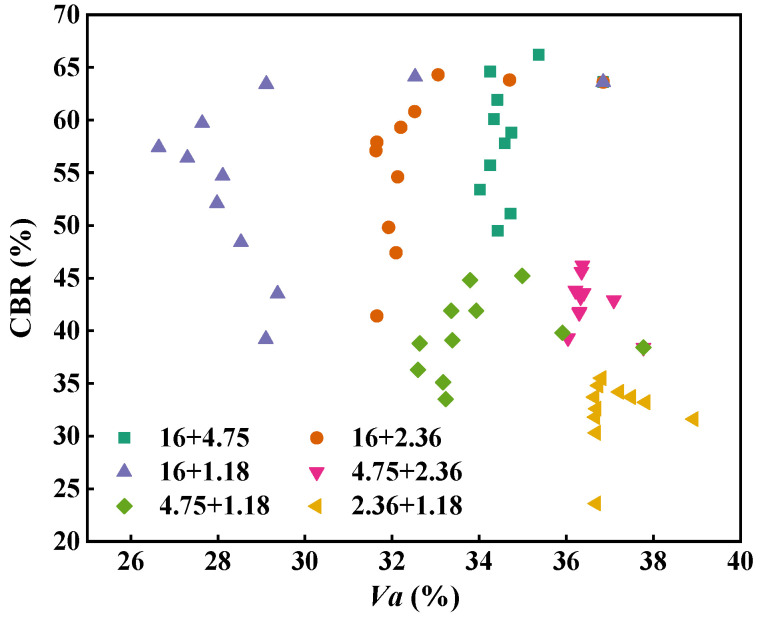
Relationship between the air void and CBR for the two-size aggregate blend.

Figure 16 depicts the relationship between the air void and CBR for the two-size aggregate blend. As can be seen, for the two-size aggregate blend, there is no obvious correlation between the air void and CBR value.

**Figure 17:**
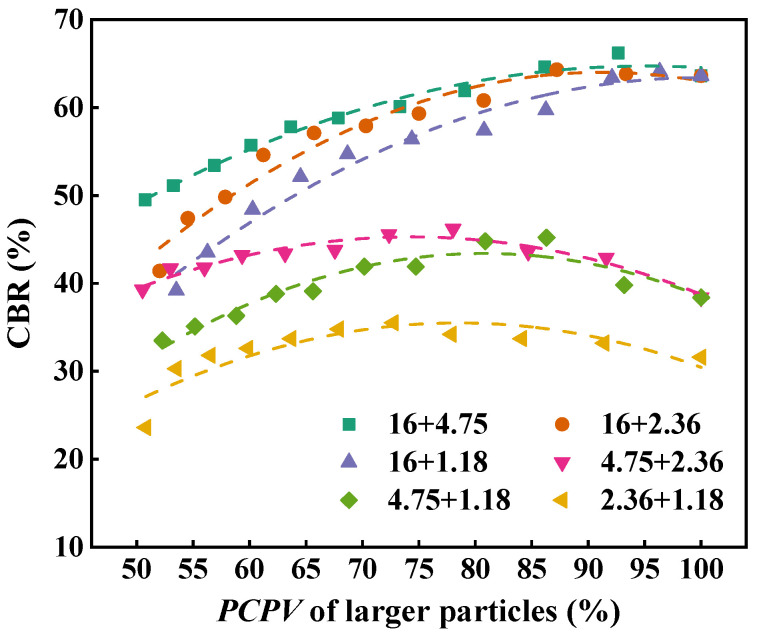
Relationship between the PCPV of larger particles and CBR for the two-size aggregate blend.

Figure 17 depicts the relationship between the PCPV of larger particles and CBR for the two-size aggregate blend. Table 4 lists the curve fitting results of the PCPV of larger particles versus CBR value. As shown in Figure 17 and Table 4, the PCPV of larger particles and the CBR value show a quadratic relationship for all blends, and the correlation coefficients are larger than 0.9, except 2.36 + 1.18 blend. For 16 + 4.75, 16 + 2.36, and 16 + 1.18 blends, the higher the PCPV of larger particles, the larger the CBR value. For 4.75 + 2.36, 4.75 + 1.18, and 2.36 + 1.18 blends, the CBR value slightly increases first and then shows a downward trend with the increasing PCPV of larger particles.

#### 3.3.3. Multi-Size Blends

**Figure 18:**
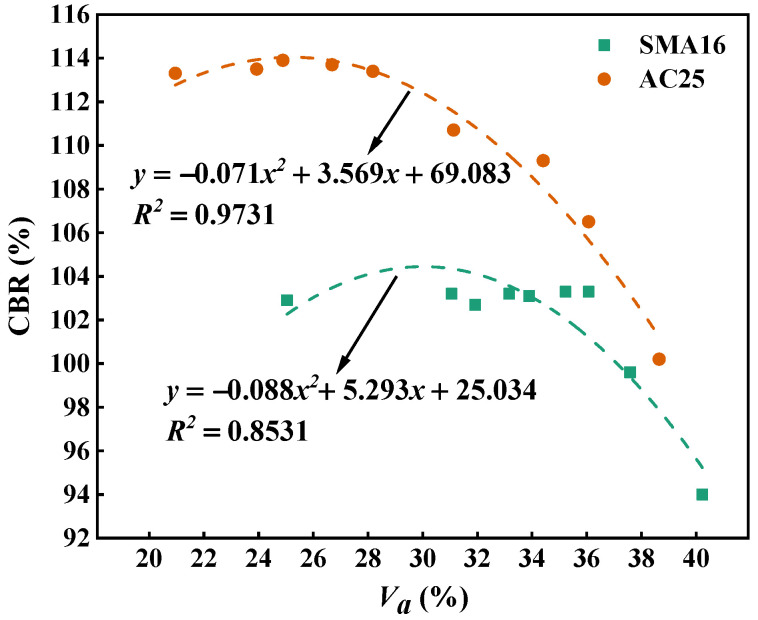
Relationship between *PCPV_s-M_* and CBR for the multi-size aggregate blend.

Figure 18 describes the relationship between air void and CBR for the multi-size aggregate blend. As can be seen, for the designed multi-size aggregate blend according to SMA16 and AC25, the air void and CBR show a quadratic relationship, and the correlation coefficients are larger than 0.85. In general, the larger the air void, the smaller the CBR value. This indicates that the air void has a significant influence on the mechanical performance of the multi-size blend. In addition, the CBR values of AC25 blends are larger than those of SMA16 blends. The reason for this is that there are more coarse aggregates in AC25 blends compared with SMA16 blends, which can provide stronger penetration strength for aggregate blends.

## 4. Conclusions

In this study, the single-size, two-size, and multi-size aggregate blends were selected to conduct the laboratory packing and CBR test. The packing characteristics and mechanical performance of aggregate blends were investigated. The relationships between the packing characteristics and mechanical performance were analyzed. The main conclusions are as follows:

(1) The particle size and the size ratio have an effect on the packing characteristics of aggregate blend. The smaller the particle size, the larger the air void of the blend. For the two-size blends, the larger the size ratio, the greater the air void of the blend and the faster the decrease in the air void with the increase in the mass percentage of smaller particles. The addition of smaller particles in larger particles can reduce the air void of the blend. The PCPV of larger particles shows a decrease trend with the increasing mass percentage of smaller particles in two-size aggregate blend.

(2) Particles smaller than 1.18 mm and those smaller than 0.3 mm in AC25 mainly play a role to fill the voids and have little contribution to the load bearing. The air voids of the designed multi-size blends according to AC25 decrease more quickly with the addition of smaller particles than those of the designed multi-size blends according to SMA16. For the multi-size blends, with the addition of smaller particles, the change in *PCPV_s-M_* can be divided into two stages: increasing stages and steady stages. The *PCPV_s-M_* in SMA-16 grows faster than that in AC25, indicating that the composition ratio of particles with different sizes has an effect on the PCPV of the blend.

(3) The blends composed of larger particles have better load bearing capacity than those composed of smaller particles. For the two-size blends, with the increase in the mass percentage of smaller particles, the CBR value of the blend slightly increases first and then gradually decreases, indicating that the mechanical performance and bearing capacity of the blend can be improved by properly increasing the smaller particles in larger particles. The larger the particle size ratio, the weaker the load bearing capacity. For the multi-size aggregate blend, the CBR values of the blends increases first and then keeps stable with the addition of smaller particles. The designed blends according to AC25 have larger CBR compared with those according to SMA16, indicating that the load bearing capacity of AC25 is better than that of SMA16.

(4) For the single-size aggregate blend, the larger the air void, the smaller the CBR value, and the air void and CBR show a significant quadratic relationship. For the two-size aggregate blends, there is no obvious correlation between the air void and CBR value, but the PCPV of larger particles and CBR value show a quadratic relationship. For the designed multi-size aggregate blend according to SMA16 and AC25, the air void and CBR value show a quadratic relationship, and the *PCPV_s-M_* and the CBR value show a good linear relationship.

The significance of this study is in providing a reference to design and optimize gradations. Therefore, aggregate blends with various compositions were considered. This research presents preliminary findings about the mechanical properties and packing features of aggregate blend. In the future study, the mesoscopic mechanical and structural characteristics of asphalt mixture need to be further explored by DEM and DIP technique. Although the CBR value provides a reasonable way to assess the material performance, it is only a relative strength. In the future study, the dynamic triaxial test can be conducted to analyze the mechanical behavior of aggregate blends.

## Figures and Tables

**Table 1:** Properties of aggregates and filler.

Size (mm)	Bulk Density (g/cm^3^)	Los Angeles Abrasion	Water Absorption (%)	Hydrophilic Coefficient	Water Content (%)
26.5–31.5	2.854	≤28%	0.450	/	/
19–26.5	2.928		0.438	/	/
16–19	2.892		0.445	/	/
13.2–16	2.829		0.462	/	/
9.5–13.2	2.764		0.600	/	/
4.75–9.5	2.762		0.718	/	/
2.36–4.75	2.669	/	0.430	/	/
1.18–2.36	2.622	/	1.312	/	/
0.6–1.18	2.601	/	1.526	/	/
0.3–0.6	2.629	/	1.127	/	/
0.15–0.3	2.599	/	1.251	/	/
0.075–0.15	2.567	/	1.691	/	/
Filler	2.802	/	/	0.7	0.33

**Table 2:** The designed aggregate blends.

Aggregate Blend	Composition
Single-size	*A*_16_, *A*_13.2_, *A*_9.5_, *A*_4.75_, *A*_2.36_, *A*_1.18_
Two-size	16 + 4.75, 16 + 2.36, 16 + 1.18, 4.75 + 2.36, 4.75 + 1.18, 2.36 + 1.18 (The mass ratios of the smaller particles to larger particles are, respectively, 1:10, 2:10, 3:10, 4:10, 5:10, 6:10, 7:10, 8:10, 9:10, and 10:10, i.e., the mass percentages of the smaller particles are, respectively, 9.1%, 16.7%, 23.1%, 28.6%, 33.3%, 37.5%, 41.2%, 44.4%, 47.4%, and 50%)
Multi-size	*A_9.5-M_*, *A_4.75-M_*, *A_2.36-M_*, *A_1.18-M_*, *A_0.6-M_*, *A_0.3-M_*, *A_0.15-M_*, *A_0.075-M_*, *A* (The composition ratio of each blend was determined according to SMA16 and AC25)

**Table 3:** Standard pressure of CBR test.

Penetration depth (mm)	2.5	5.0	7.5	10.0	12.5
Standard pressure (MPa)	7.0	10.5	13.4	16.2	18.3

**Table 4:** Curve fitting results of the PCPV of larger particles versus CBR value.

Blend Type	Fitting Equations	*R^2^*
16 + 4.75	*y* = (−0.008 ± 0.001)*x*^2^ + (1.464 ± 0.181)*x* – (4.933 ± 6.52)	0.98
16 + 2.36	*y* = (−0.013 ± 0.002)*x*^2^ + (2.373 ± 0.363)*x* – (44.25 ± 13.24)	0.96
16 + 1.18	*y* = (−0.01 ± 0.002)*x*^2^ + (2.057 ± 0.378)*x* – (39.57 ± 14.02)	0.97
4.75 + 2.36	*y* = (−0.01 ± 0.001)*x*^2^ + (1.478 ± 0.157)*x* – (9.673 ± 5.64)	0.92
4.75 + 1.18	*y* = (−0.013 ± 0.002)*x*^2^ + (2.136 ± 0.294)*x* – (42.89 ± 10.76)	0.91
2.36 + 1.18	*y* = (−0.011 ± 0.002)*x*^2^ + (1.716 ± 0.368)*x* – (31.86 ± 13.26)	0.77

Note: *x* and *y* represent the PCPV of larger particles and the CBR value of the corresponding aggregate blend, respectively.

## Data Availability

The original contributions presented in this study are included in the article. Further inquiries can be directed to the corresponding authors.
